# Pathogenic bacteria enhance dispersal through alteration of *Drosophila* social communication

**DOI:** 10.1038/s41467-017-00334-9

**Published:** 2017-08-16

**Authors:** Ian W. Keesey, Sarah Koerte, Mohammed A. Khallaf, Tom Retzke, Aurélien Guillou, Ewald Grosse-Wilde, Nicolas Buchon, Markus Knaden, Bill S. Hansson

**Affiliations:** 1 Department of Evolutionary Neuroethology, Max Planck Institute for Chemical Ecology, Beutenberg Campus, Hans-Knöll-Straße 8, D-07745 Jena, Germany; 2 Department of Entomology, Cornell University, 5124 Comstock Hall, Ithaca, NY 14853 USA

## Abstract

Pathogens and parasites can manipulate their hosts to optimize their own fitness. For instance, bacterial pathogens have been shown to affect their host plants’ volatile and non-volatile metabolites, which results in increased attraction of insect vectors to the plant, and, hence, to increased pathogen dispersal. Behavioral manipulation by parasites has also been shown for mice, snails and zebrafish as well as for insects. Here we show that infection by pathogenic bacteria alters the social communication system of *Drosophila melanogaster*. More specifically, infected flies and their frass emit dramatically increased amounts of fly odors, including the aggregation pheromones methyl laurate, methyl myristate, and methyl palmitate, attracting healthy flies, which in turn become infected and further enhance pathogen dispersal. Thus, olfactory cues for attraction and aggregation are vulnerable to pathogenic manipulation, and we show that the alteration of social pheromones can be beneficial to the microbe while detrimental to the insect host.

## Abstract (web summary)

Behavioral manipulation of host by pathogens has been observed in vertebrates, invertebrates, and plants. Here the authors show that in *Drosophila*, infection with pathogenic bacteria leads to increased pheromone release, which attracts healthy flies. This process benefits the pathogen since it enhances bacterial dispersal, but is detrimental to the host.

## Introduction

Certain pathogens, parasites, and viruses possess the ability to manipulate their host, including examples in vertebrates^1–3^, invertebrates^4–7^ as well as in plants^8–10^. For instance, bacterial pathogens use several strategies to hijack plant host physiology to their own benefit while often to the detriment of their host plant, including alterations of volatile and non-volatile host metabolites and immune-related proteins. This change in volatile release after host–plant infection can also lead to an enhanced attraction of insect vectors to the infected plant, and can therefore lead to increased pathogen dispersal by insect vectors^8, 9, 11^. It has also been shown that a pathogenic bacterium, *Pseudomonas syringae*, is able to alter the physiology of its plant host, *Arabidopsis*, in order to enhance bacterial growth and to help the bacterium avoid defensive responses within the host by altering hormone signaling as well as host susceptibility^10^. In the case of the parasitic flatworm, *Leucochloridium paradoxum*, it infects land snails and the parasite congregates in the eye stalks, where it pulsates different colors and patterns in a display to make the snail more noticeable to bird predators, which are the primary host of this flatworm^6^. Similarly, rats and mice lose their fear of cats upon infection with the parasite *Toxoplasma gondii* and subsequently become more likely to be killed and consumed by a cat, again the primary host of the parasite^12^. This fearless or suicidal behavior in mice has subsequently been shown to be due to an impairment of the olfactory receptors that usually trigger aversion to feline urine, and that this olfactory impairment is caused directly via the infection by the *Toxoplasma* parasite^1, 13^. Other systems for the study of pathogenic alteration of behavior include several examples within insect hosts, such as ants^5, 14^, crickets^4^, and leafhoppers^11^. Thus, in both plants and animals, microorganisms have been shown to alter the behavior and physiology of a host in order to provide a benefit to the pathogen. However, especially in animal systems, the specific mechanisms for host alteration by pathogens and parasites are not well understood.

*Drosophila* has been a powerful model to study bacterial infection as it pertains to immune, hormonal, and metabolic responses mounted by the insect host^15–18^. Several strains of pathogenic bacteria, including *Erwinia carotovora* sp*. carotovora* 15 (Ecc15), *Serratia marescens Db11*, and *Pseudomonas entomophila*, have been well characterized in regard to the immune responses elicited by *Drosophila melanogaster* following infection^15, 19–22^, and thus these bacteria have arisen as a part of a model system for the study of insect immunity. Although *D. melanogaster* does not possess an adaptive immune system, their innate immune defense has proven to be efficient against most bacteria that are ingested or injected into the fly, perhaps an evolutionary result of living and breeding in high-density, and within microbe-rich food substrates such as rotten and decaying fruit^17, 23^. The *Erwinia* bacterium we use in this study is a member of the Gram-negative Enterobacteriaceae family, several species of which are phytopathogenic, often causing soft rots on fleshy fruits, vegetables, and ornamental crops^24, 25^. This bacterial pathogen has developed sustained plant-to-plant infection cycles, usually via insect vectors such as Hymenopterans and Dipterans^24, 25^. This bacterium also overlaps with the preferred host range of *D. melanogaster*, an insect that has a strong preference for decaying or rotting substrates. Moreover, *D. melanogaster* has been previously shown to be a natural vector for *Erwinia carotovora carotovora* and *E. carotovora atroseptica*, both of which cause potato blackleg disease. *Drosophila* are found naturally carrying these strains of bacteria in potato fields, and, at least under greenhouse conditions, it has been established that the vinegar fly is able to vector blackleg disease between potato plants^26, 27^. Similarly, *P. entomophila* was originally described from field-collected *Drosophila* ^20^; thus, fly infection by this bacteria is also thought to be naturally occurring. In addition, the strain of *S. marcescens* we use is highly pathogenic to *D. melanogaster*, and one which has been described from these insects^21^; moreover, bacterial community surveys in natural field conditions have demonstrated that Enterobacteriaceae, including the genus *Serratia*, are found naturally in the wild and within naturally occurring populations of *Drosophila* ^28^. Therefore, we can hypothesize that the activation of the *Drosophila* immune response by certain strains of bacteria indicates that these bacteria have some natural interaction with the fly, and that these bacteria can perhaps exploit *Drosophila* as a potential intermediate host as well as a vector between fruits, vegetables, or other plants. We also tested other naturally occurring, non-pathogenic bacteria, such as *Acetobacter pomorum* and *Lactobacillus plantarum*, neither of which have been shown to induce substantial immune responses, and are the dominant bacteria strains within the midgut and hindgut of *D. melanogaster* adults and larvae^29^.

In previous studies, the ability of *Drosophila* to detect and avoid potentially harmful microorganisms in their environment has been elucidated, such as for pathogenic fungi and bacteria^30–33^. These studies have outlined two olfactory (geosmin, Or56a; phenol, Or46a) and a single gustatory avoidance pathway (lipopolysaccharides, Gr66a) that allow the fly to avoid certain pathogens when presented alone. Conversely, and counter to our initial hypotheses, here we show for the first time that flies become strongly attracted toward conspecifics that have become infected by specific pathogenic bacteria. Moreover, we demonstrate that the increased attraction toward infected flies is due to amplified aggregation pheromone emission by infected flies and their feces, and that this increase is mediated by pathogen-induced alterations to immune, hormonal, and metabolic response cascades following infection.

## Results

### Behavioral response toward sites of infection

**Fig. 1:**
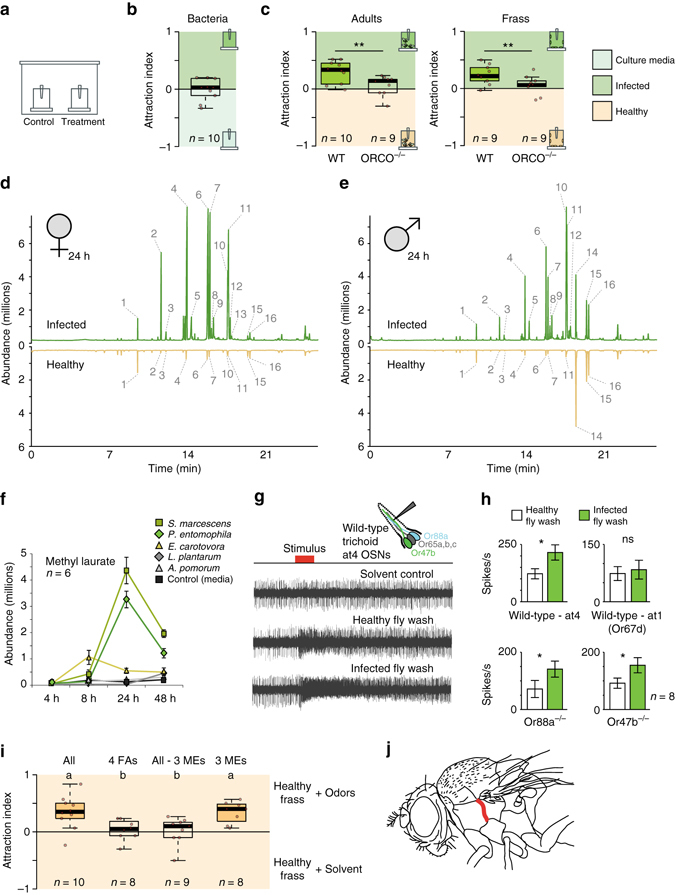
Effects of infection on *Drosophila* attraction and odor profile. **a** Experimental design of attraction assays. **b** Attraction index of adult *Drosophila* toward the olfactory cues from *Pseudomonas* bacteria or from growth media control. **c** Attraction indices or naive wild type or Orco mutant flies given the choice between other adults with and without *Pseudomonas* infection or between frass of flies with or without infection. Attraction index: ((no. of flies in treatment trap) − (no. of flies in control trap)) / total no. of flies. **d**, **e** GC-MS profile of female **d** and male **e** *Drosophila* adults either infected with *Pseudomonas entomophila* bacteria or mock-infected with growth media (healthy control). Numbers from GC-MS refer to FID peaks: (1) bromodecane (internal standard); (2) methyl laurate; (3) lauric acid; (3) methyl myristate; (5) myristic acid; (6) methyl palmitoleate; (7) methyl palmitate; (8) palmitoleic acid; (9) palmitic acid; (10) methyl linoleate; (11) methyl oleate; (12) methyl stearate; (13) oleic acid; (14) Z-11-cis-vaccenyl actetate (cVA); (15) 7-Z-tricosene; (16) heneicosane. **f** Amount of methyl laurate produced over time, from 4 to 48 h after infection with several strains of bacteria (for time courses of other compounds see Supplementary Fig. 2D). **g** Example of SSR responses of healthy *Drosophila* antennal trichoid (at4) neurons to body washes of infected or healthy *Drosophila*. Stimulus: 0.5 s. **h** Quantified SSR responses toward healthy or infected fly body washes, including recordings from wild-type at4 and at1 neurons, as well as from fly mutants for Or47b and Or88a pheromone OSNs. **i** Attraction indices of adult *Drosophila* toward healthy frass perfumed with treatment odors or solvent control. Frass was perfumed either with all odors (All) that were increased after infection or with a subset. 4FAs: mixture of fatty acids increased after infection that were reported to act as pheromones (lauric acid, myristic acid, palmitoleic acid, and palmitic acid, Lin et al.^36^ ); 3 MEs: methyl esters (ML, MM, and MP) increased after infection and reported to act as pheromones (Dweck et al.^35^). More details in Supplementary Fig. 2A. **j** Schematic of septic or systemic infection location for both bacterial and mock infection. *Filled boxes* denote significance from zero

We first tested the behavioral response of *Drosophila* in attraction, feeding, and oviposition toward a natural pathogen, the bacterium *P. entomophila* (Fig. 1a–c and Supplementary Fig. 1A–G). While flies did not respond to the odor of *P. entomophila* in an attraction assay (Fig. 1a, b), we could confirm previous findings from Soldano et al. that flies avoid feeding and ovipositing on food sources containing Gram-negative bacterial pathogens (Supplementary Fig. 1A, C). However, we were also interested in whether *Drosophila* can identify and avoid infected conspecifics as these individuals could be another potential source of infection within the population. Therefore, we repeated the behavioral assays but did not present the pathogen alone, but instead tested infected flies or their feces (Fig. 1c). While both oral and systemic infection generated similar results, for consistency, and to ensure similar levels of infection, all flies were systemically infected along the pleural suture line along the mesothorax with growth media containing bacteria or mock infected with growth media only as a control (Fig. 1j). Contrary to our initial expectation, *Drosophila* strongly preferred the odor of infected flies (or feces of infected flies) over that of healthy flies (or their feces) in the attraction assays (Fig. 1c). We repeated these tests of attraction using an alternative behavioral paradigm, and again we were able to observe that flies were significantly more attracted toward the odors from infected flies when compared to those of healthy controls (Supplementary Fig. 1E). In tests with Orco mutant flies, this preference for infected conspecifics and their feces was lost; thus, we concluded that this attraction was due to olfactory cues (Fig. 1c). We gained similar results when we tested the body washes of infected flies or their feces in feeding and oviposition assays (Supplementary Fig. 1b, d). In both cases the flies avoided the bacterium when it was presented alone; however, the flies did not avoid sites of infection and instead preferred infected individuals and material generated by the infected flies (Fig. 1c and Supplementary Fig. 1B, D). Interestingly, the oviposition-related attraction of infected flies was time-sensitive and peaked between 16 and 24 h after infection, while the infected flies were still alive, but dropped after their death (i.e., 48 h after infection, Supplementary Fig. 1D). Thus, it seems that the repulsive behavioral effect of pathogenic bacteria when presented alone can be overcome by the attractive odors generated by infected flies and their feces.

### Insect-derived odor emission following infection

In order to examine any odor-derived differences between healthy and infected *Drosophila*, we performed extensive gas chromatography mass spectrometry (GC-MS) analyses of the volatile and non-volatile chemical cues associated with *Drosophila* following systemic infection with pathogenic and non-pathogenic strains of bacteria. While infection with the non-pathogenic *L. plantarum* or *A. pomorum*, or with the facultative endosymbiont *Wolbachia* ^34^, did not generate any significant difference in the odor profile of the fly (Supplementary Fig. 2C), infection with three strains of natural bacterial pathogens, including *S. marcescens*, *E. carotovora carotovora* (*Pectobacterium carotovora*), and *P. entomophila*, each induced large changes in the chemical profile of both sexes of infected flies (as compared to mock-infected controls) (Fig. 1d–f; Supplementary Fig. 2C, D). This increase in fly odors after infection included in total 12 compounds (Supplementary Fig. 2A, B). Interestingly, after infection, many of the 12 compounds for which emission increased significantly have been previously identified as *Drosophila* pheromones that modulate courtship and aggregation^35, 36^, including methyl laurate (ML), methyl myristate (MM), methyl palmitate (MP), and palmitoleic acid (PA). However, notably, *cis*-vaccenyl acetate (cVA), the male-specific pheromone produced by the male accessory glands, was not affected by any tested bacterial infection (Fig. 1e).

To further examine the increase in pheromone production after infection, we next quantified the amount released over time (Fig. 1f and Supplementary Fig. 2D). After systemic infection with *E. carotovora*, pheromone production peaked around 8 h post infection and returned thereafter to normal levels comparable to those found in control or mock-infected flies. Infection with this strain of bacteria is non-lethal, as the vinegar flies are able to mount a successful immune response to thwart the infection^15^. However, in the case of both *P. entomophila* and *S. marcescens*, pheromone production continued to increase dramatically until the death of the fly, usually around 24 h post infection, with pheromone levels in dead flies then decreasing rapidly toward control levels (Fig. 1f and Supplementary Fig. 2D).

### Olfactory response to odors from healthy and infected flies

After having established that pheromone production was highly upregulated in live flies following infection with specific pathogenic bacteria, we proceeded to investigate differences in olfactory responses to this increase in the odor profile of the fly. Using single sensillum recordings (SSRs), we could demonstrate that healthy *D. melanogaster* flies show an increased olfactory response to body washes from infected flies when compared to that of healthy flies (Fig. 1g). We could also show that this response is elicited from olfactory sensory neurons (OSNs) present in the at4 but not in the at1 sensillum (Fig. 1h), and, more specifically, elicited by ligands of the olfactory receptors Or47b and Or88a (i.e., ML, MM, and MP^35^; Fig. 1g, h and Supplementary Fig. 3A–D). Notably, despite PA and several other fatty acids being increased for flies infected with *P. entomophila*, these suggested Or47b ligands^36^ did not activate any of the tested OSNs within the at4 sensillum (Supplementary Fig. 3A–D), nor did any of these fatty acids generate a preference in *Drosophila* behavior (Fig. 1i). Together, these results match our previous GC-MS analyses that showed increases after infection for fatty-acid-derived ligands (detected in at4 trichoid sensillae) but not in cVA (detected in at1 sensillum). Moreover, we could show that three fatty-acid methyl esters (ML, MM, and MP) were necessary and sufficient to account for the increased behavioral attraction and electrophysiological response following infection of *Drosophila* with *P. entomophila* bacteria (Fig. 1I and Supplementary Fig. 3A–D).

### Pheromone changes with immune and metabolic cascades

**Fig. 2:**
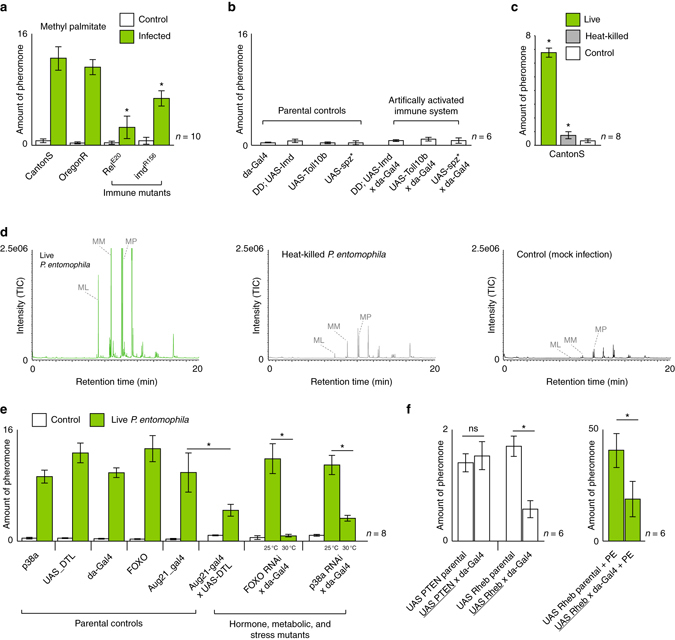
Pheromone production after infection with immune and metabolic mutants. **a**, **b** Pheromone (here and thereafter methyl palmitate shown as example) production of CantonS or OregonR wild-type flies, and flies deficient in the Imd immune response pathway (RelE20 and IMD^156^) with *Pseudomonas entomophila* infection (*green*) and without (*white*; **a**), or of flies, where the IMD (Imd flies) or the Toll (Toll flies and spz* flies) response pathway were artificially activated (**b**). **c** Pheromone production after infection with live (*green*) or heat-killed (*gray*) bacteria. **d** GC-MS profiles of live bacteria, heat-killed bacteria, and mock-infected control flies. **e** Pheromone production of flies with either decreased juvenile hormone (Aug21-gal4 x UAS-DTI flies), insulin metabolism (FOXO flies), or stress responses (p38a flies) and their parental control lines. RNAi lines tested at the non-active 25 °C served as further controls. **f** Pheromone production of flies with or without artificial activation of Rheb (an inhibitor of the FOXO pathway) with infection (*green*) or without (*white*)

Since the pheromone production over time closely matches the published timeline of the immune response to infection for *E. carotovora* and *P. entomophila* ^15, 20^, we next focused on repeating the GC-MS experiments with immune, hormonal, and metabolic *D. melanogaster* mutants in order to identify any involvement of these pathways in the increased production of pheromones following infection by these bacterial pathogens. Healthy flies with a reduced immune induction (e.g., Rel^E20^ and Imd^R156^ flies)^37^ produced normal amounts of pheromones relative to Canton S, but following infection, the same flies produced significantly less pheromones compared to infected wild type (WT) and other control flies (Fig. 2a). This suggests that a functional Imd pathway is necessary for the increase in pheromone production following infection. Moreover, we found that impairment of either the Imd or the Toll immune response pathway resulted in a lower maximum amount of pheromone production after infection with *P. entomophila* (Supplementary Fig. 4A). However, when we tested flies that had either their Imd or Toll immune response pathways artificially activated in the absence of bacteria, we could not induce this increase in pheromones (Fig. 2b), suggesting that the immune system is necessary but not sufficient to account for the change in pheromone production following *P. entomophila* infection. Infection with dead, but intact bacteria can still result in an immune response, including the increase of antimicrobial peptides (AMPs) such as diptericin and drosomycin^15, 19, 38^. We therefore tested whether an infection with heat-killed *P. entomophila* was sufficient to yield AMPs (Supplementary Fig. 4B). Although heat-killed bacteria resulted in the production of two different AMPs and a smaller but significant increase in pheromone production, infection with heat-killed bacteria never reached the degree of pheromone production observed in flies infected with living bacteria (Fig. 2c, d). This suggests that ongoing bacterial growth and subsequent damage by the pathogen are required to induce the large increases in pheromone production.

In addition to the immune response, the fly hormonal system as well as metabolic and stress responses are also affected by bacterial infection, especially in relation to the utilization of the fat body, inflammation, and the mobilization of energy to combat infection, which primarily results in a decrease in adult fat body content^16, 39^. With this in mind, we next focused on the potential origin of these fatty-acid pheromone odors (ML, MM, and MP) by using transgenic fly lines that were deficient in their ability to synthesize juvenile hormone (Aug21-Gal4 > UAS-DTI), flies that were deficient in the transcription factor FOXO (a transcription factor related to insulin signaling and induced in response to stress, pathogenic damage, and starvation), as well as flies deficient in the stress response pathway regulator p38a. Alterations of some of these pathways can be lethal during fly development; thus, in these cases we took advantage of RNA interference (RNAi) inducibility to pass fly development and still test the function of otherwise lethal genes in adult *Drosophila*. The reduction of juvenile hormone through the UAS-Gal4 system, or FOXO via RNAi, caused a significant decrease in pheromone production after infection when compared to the parental lines or to the genetically identical RNAi controls that had not been activated by temperature (Fig. 2e). As p38 directly phosphorylates FOXO^40^, these two mutants have already been shown to be linked in their function. Hence, by repeating the experiments with p38a RNAi flies, we were able to confirm the involvement of FOXO in the increased pheromone production after infection. As inhibiting the FOXO transcription factor (either directly through FOXO RNAi or indirectly through p38a RNAi) revealed the most drastic reduction in pheromone production after infection (Fig. 2e), we next activated the *Rheb* gene (part of the target of rapamycin signaling pathway, and which is an inhibitor of the product of FOXO)^17, 41, 42^. As we expected, activating Rheb (and by that indirectly decreasing the product of FOXO), we again found a significant decrease in pheromones, even in the absence of any infection, as well as a strong decrease in infected flies relative to the infected controls (Fig. 2f), thus reconfirming the involvement of FOXO in the pathogen-induced pheromone production. However, when we tested flies in which we increased the expression of PTEN, a factor that is only distantly related to the FOXO transcription factor within the insulin receptor pathway (InR), we did not find any effect on pheromone production (Fig. 2f). Hence, it appears that several but not all genes related to this metabolic cascade may be influenced by *P. entomophila* infection. When testing oviposition with body washes of flies that were either deficient in their immune response (Relish) or metabolic response (FOXO), we observed a reduced preference for infected flies (Supplementary Fig. 1H). As both immune (Relish) and insulin response pathway mutants (FOXO) resulted in reduced pheromone production after infection, and a corresponding decrease in behavioral preference following infection (compared to WT-infected flies), we conclude that both of these general signaling cascades (immunity and insulin metabolism) are required for *P. entomophila* to alter the fatty-acid pheromone production of *D. melanogaster* adults.

### Ecological effects of pheromone changes after infection

**Fig. 3:**
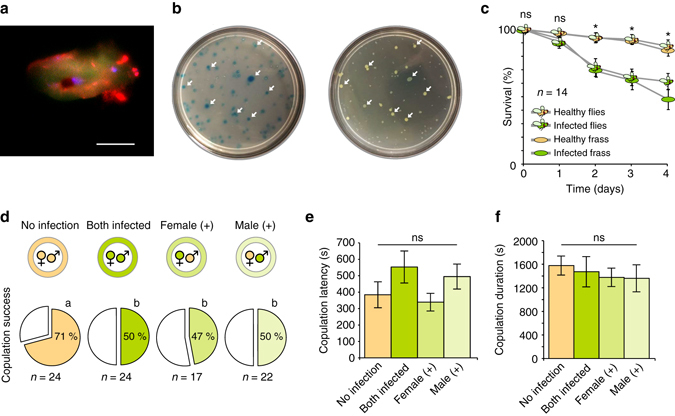
Ecological impact of preference for infected flies and frass. **a** Frass droplet from a fly that was fed green fluorescent protein (GFP)-labeled bacteria, showing live bacteria (*green*) and dead bacteria (*red*) present in the feces. *Scale bar* depicts 10 µm. **b** Flies fed with a solution containing bacteria and blue dye were allowed to deposit frass onto an agar plate (*left*), and bacterial colony growth was assessed from the fecal deposits (*right*), demonstrating that bacteria can survive the digestive tract and be transferred via feces to new locations. **c** Survival over time of cohorts of flies reared in containers that held either healthy or infected fly adults or their frass (see Supplementary Fig. 5). **d**–**f** Copulation success (**d**), latency (**e**), and duration (**f**) of single pairs of flies following all combinations of infection

We next examined the potential costs and benefits of increased pheromone production for both the insect and the bacteria. Our analyses of fecal material using green fluorescent protein-labeled bacteria revealed that ingested bacteria can survive the digestive tract (Fig. 3a), which was similar to studies that confirmed that yeast can survive ingestion by *Drosophila* and be passed through fecal deposits^43^. In addition, by using blue dye in feeding solutions, we could show that frass deposited on agar plates by infected flies (Fig. 3b, *left*) resulted in new bacterial colonies at the same locations (Fig. 3b, *right*), providing further support that pathogenic bacteria can survive passage through the *Drosophila* digestive system and be transferred to new locations via the oral–fecal route. To study the transmission of bacteria through infected frass material, we introduced healthy flies to containers that held infected conspecifics or to containers in which flies were removed but their frass remained (Supplementary Fig. 5A–D). In both cases we could observe an acute increase in the mortality of the introduced flies when in the presence of infected conspecifics or infected frass (Fig. 3c). Thus, the *P. entomophila* pathogen survives the *Drosophila* gut and potentially profits from increased contact and dispersal through increased attraction of healthy flies toward infected flies or their frass, material that has been previously shown to be attractive for *Drosophila* adults^44^. Moreover, this attraction to infected flies has a high cost for the arriving flies, as they run an increased risk of becoming infected and dying. Conversely, the same attraction could be beneficial for the infected flies, as it could increase their chances of finding a mate and reproducing before death. Thus, we conducted mating assays where all combinations of healthy and infected flies were tested (Fig. 3d). When we presented a healthy and an infected female to two males, preliminary experiments indicated increased orientation and courtship displays toward the infected female; however, in single-pair mating experiments, infection always resulted in lower copulation success, irrespective of whether the female, the male, or both flies were infected (Fig. 3d–f). We furthermore found that infected females were less likely to accept any male for copulation, as they were less likely to extend their abdomen or separate their wings during the male courtship song. We thus found no benefit to the infected fly with regard to successful copulation, even given the robust increase in pheromone production, perhaps due to other confounding behavioral alterations after infection, such as lethargy, cell damage, or another byproduct of pathogen growth. Although the increased pheromone emission did not result in the hypothesized higher mating success of infected flies, we cannot exclude that without this increase infected flies would even have less copulation. It is also possible that different degrees of infection may result in increased courtship success, although additional work is required to address this hypothesis. Therefore, our current data suggest that the increased pheromone emission of infected flies mainly results in attracting more *Drosophila* to sites of infection, promoting contact and dispersal benefits for the bacterial pathogens, while not providing any direct courtship benefit to the infected fly.

### Pathogenic infection with other Dipterans

**Fig. 4:**
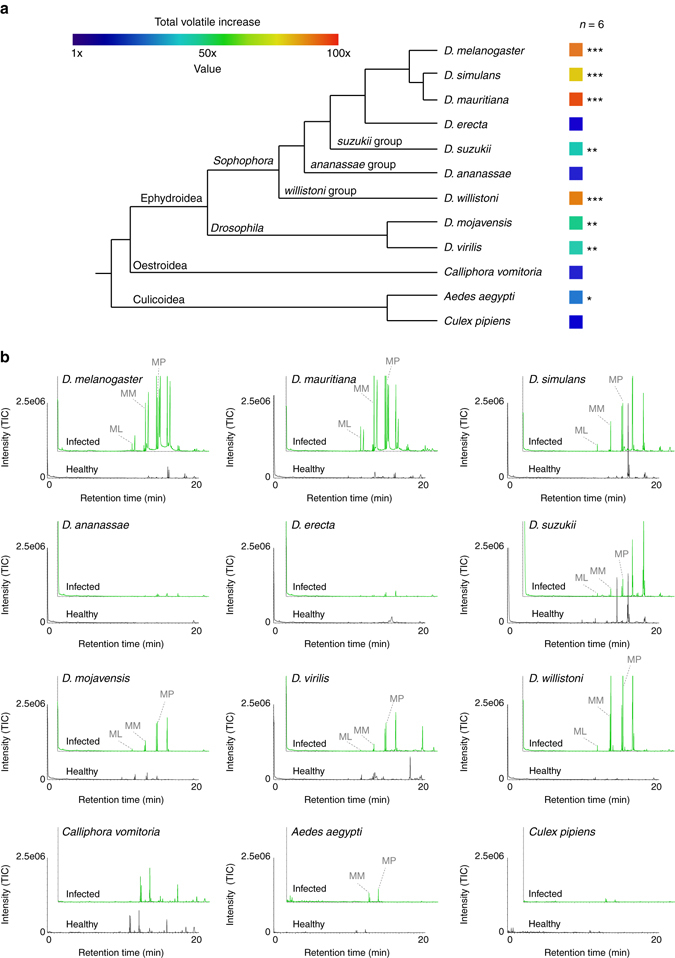
Infection of *Drosophila* and Diptera species with *P. entomophila* bacteria. **a** Phylogenetical relationship and color-coded relative increase of odor emissions after infection for all tested species. **b** Example of GC-MS traces for each species before and after infection with *P. entomophila* bacteria with those methyl esters identified that were behaviorally relevant in *D. melanogaster*

To augment our screening of *D. melanogaster*, we also tested *P. entomophila* infection with eight other Drosophilids and three other Dipterans, including the blue bottle fly, *Calliphora vomitoria*, as well as two mosquitoes, *Aedes aegypti* and *Culex pipiens* (Fig. 4a, b). While infections were lethal for all tested insect species, we found significantly increased emissions of potential fatty-acid pheromones in seven out of nine *Drosophila* species as well as in *A. aegypti* (but no increase in *Calliphora* nor in *Culex*), suggesting that the manipulation of the insect’s volatile emission by the pathogen *P. entomophila* is a more general phenomenon.

## Discussion

We conclude that specific pathogenic bacteria can overcome the avoidance mechanisms of *D. melanogaster* flies^32^ by taking advantage of, or hijacking, a chemosensory circuit related to social communication^35, 36, 45^. This preference and attraction toward infected individuals is due to pheromone signals and cannot be avoided by conspecific flies, as these chemical cues are vital for both aggregation and courtship in *D. melanogaster*. While previous research has documented viral or parasitic alterations in pheromone production for *Helicoverpa zea* and *Apis mellifera* ^7, 46^, the ecological impact as well as physiological and neural mechanisms for this shift have not been previously addressed. Here we assert that both the immune response pathway and the InR pathway are necessary for this increase in fatty-acid-derived pheromone release after infection by *P. entomophila* bacteria. This linkage between the *Drosophila* immune system, insulin signaling, and the fat body has been previously noted^39^, as has the connection between the Rheb, FOXO, and damage response pathways^41^. However, our data show for the first time a pheromone change in *Drosophila* after infection, and show a mechanistic connection between the pathogen and the alteration of the pheromone communication system of the insect host. In addition, our data also reveal for the first time the associated ecological ramifications for both the pathogen and for the insect following infection.

This increase in pheromone production after infection might just be a byproduct of the bacterial growth and the associated damage to the insect^16, 39^; however, this insect–microbe interaction results in a potential evolutionary advantage for the bacterium by increasing its chances for contact and dispersal through enhancing several aggregation pheromones of a potential host and insect vector. Previously, it has been suggested that humans infected with malaria are more attractive to the *Anopheles* vector and that mosquito vectors carrying Malaria are also more likely to take additional bloodmeals, both of which result in increased dispersal benefits for the *Plasmodium* protozoan^47–50^. Our data may be pertinent for not only the study of insect-transmitted human diseases, but also studies related to insect-vectored plant pathogens, such as those similar to the *Drosophila-*transmitted plant pathogen *E. carotovora* used in this study. In addition, the application of species-specific pathogens may be useful as a tool in identifying novel pheromones from other infected host organisms, such as *D. suzukii* or *A. aegypti*.

Therefore, in summary, it is our assertion that specific pathogenic bacteria alter the lipid metabolism of *Drosophila* during infection through both immune and insulin signaling pathways, which results in increased fatty-acid pheromone release by the adult insect after infection. Moreover, this increase in pheromone release attracts more adult flies to sites of infection and contributes to the potential uptake and dispersal of the pathogenic bacteria toward new fruit, vegetable, or insect hosts. Thus, our data begin to generate a better understanding of how microorganisms can alter insect host physiology as well as alter insect host behavior, and, moreover, our findings might provide future tools or novel strategies to combat insect-transmitted human and plant diseases.

## Methods

### *Drosophila* stocks

WT fly lines included the *D. melanogaster* Canton-S and OregonR strains. Flies were raised on standard diet at 25 °C with a 12 h light/dark cycle. Transgenic lines related to immunity, hormones, and insulin signaling were obtained where possible from the Bloomington Drosophila Stock Center (flystocks.bio.indiana.edu), and include: *p38a RNAi*, *Rel* ^*E20*^, *DD; UAS-imd*, *UAS-Toll10b*, *FOXO RNAi*, *IMD* ^*R156*^, *UAS-Rheb* (BL 9690), *Aug21-Gal4*, *UAS-DTI*, UAS-spz*, and *da-Gal4* (Gaia). Other transgenic lines include: *Or88a* mutant (Leslie Vosshall; E4365-181) and *Or47b*[3] mutant (BL 51307). All fly lines have been described previously^15, 19, 35, 37, 41^. *Drosophila* RNAi lines after crossing were kept at 30 degree (treatment) or 25 degree (as negative controls) prior to subsequent testing with infection.

### Bacterial strains and infection experiments

Bacterial strains were kept in long-term storage at −80 °C in 70% glycerol or 70% dimethyl sulfoxide (DMSO). Fresh bacterial cultures were generated daily and cultured overnight in 1000 µl lysogeny broth (LB) growth medium and grown at 29 °C and 70% humidity^51^. Adult flies between 4 and 7 days of age were pricked with a sharpened tungsten needle that had first been sterilized with ethanol and then inoculated by dipping the needle into a concentrated bacterial pellet^52^. Control flies were also pricked in the same manner, but with only LB culture medium. Flies were maintained for set time intervals at 29 °C following infection with either the bacteria or the mock control and then later used for subsequent behavioral experiments or body wash collections. To generate heat-killed samples, fresh 1 ml bacterial cultures were placed into Eppendorf tubes and then allowed to float in a water bath that was heated to 90 °C for 1 h. After cooling to room temperature, these heat-killed bacteria were then used following the previously described pricking procedures to infect the adult flies. Bacteria were also confirmed to be dead by plating them without observing any growth.

### Trap assays and FlyWalk

Trap assays were performed with 2–5-day-old flies as previously described^44, 53^. Briefly, test chambers (transparent yoghurt cups (500 ml) with 50 ventilation holes in the lid) contained a treatment and a control trap made from small transparent plastic vials (30 ml) with a cut micropipette tip (tip diameter 2 mm) inserted into a hole of the vial. Thirty flies (males and females, ratio about 1:1, 4–5 days old, starved for 24 h before the experiment) were placed in each test box. Experiments were always started at the same time of day and carried out in a climate chamber (25 °C, 70% humidity, 12-h-light:12-h-dark cycle). The number of flies in and outside the traps was counted after 24 h. Valence of the tested cuess was quantified with an attraction index (AI), calculated as: AI = (*O*−*C*)/(30), where *O* is the number of flies in the odorant trap, *C* the number of flies in the control trap, and 30 the sum of all flies tested. The resulting index ranges from −1 (complete avoidance) to 1 (complete attraction). A value of zero characterizes a neutral or non-detected odorant. FlyWalk trials were also conducted as described previously^54, 55^. In short, 15 individual flies were placed in glass tubes (0.8 cm i.d.). The glass tubes were aligned in parallel, and flies were monitored continuously by an overhead camera. *xy* positions were recorded automatically at 20 fps using Flywalk Reloaded v1.0 software (Electricidade Em Pó; flywalk.eempo.net). Experiments were performed under red LED light (peak intensity at *λ*, 630 nm). During the experiments, flies were continuously exposed to a humidified airflow of 20 cm/s (70% relative humidity, 20 °C). Flies were repeatedly presented with pulses of various olfactory stimuli at interstimulus intervals of 90 s. Stimuli (i.e., headspace of either 100 healthy or infected adult flies (50 males and 50 females)) were added to the continuous airstream and thus traveled through the glass tubes at a constant speed. The paradigm allows us to measure the stimulus-induced change of upwind speed of the tested flies.

### Feeding assays

Flies were collected and tested between the ages of 2–5 days, and included both males and females that were starved beforehand for 18–20 h with constant access to water. Flies were then cooled for 2 min at −20 °C to assist in their transfer to the behavioral arena. The capillary feeder (CAFÉ) assays utilized glass micropipettes with liquid media that were filled by capillary action and then inserted through pipette tips into the container holding the adult flies, modified from Ja et al.^56^. One capillary contained the control (5% sucrose with LB media), while the other contained the treatment (5% sucrose plus LB media and either bacteria or frass), and the volume consumed from each side was measured after a set duration of feeding. Feeding indices were calculated as (*T *− *C*)/(*T* + *C*), where *T* is the amount of food consumed from the treatment solution and *C* is the amount of food consumed from the control solution.

### Chemical analyses and SSRs

All of the synthetic odorants that were tested and confirmed were acquired from commercial sources (Sigma, www.sigmaaldrich.com, and Bedoukian, www.bedoukian.com) and were of the highest purity available. Stimuli preparation and delivery for behavioral experiments followed previously established procedures, and collection of volatile and non-volatile compounds was carried out according to standard procedures^35, 44^. GC-MS (HP5 and HP-Innowax) and TDU-GC-MS analyses were performed on all odor collections and insect body washes as described previously^35^. The NIST mass-spectral library identifications were confirmed with chemical standards where available, and the internal standard bromodecane was utilized for quantification and statistical comparisons between analyzed samples. SSR experiments were conducted as described previously^35, 44^.

### Oviposition experiments

Virgin flies were collected and separated by sex 4–5 days prior to the experiments. Before the trials, male and female virgins were allowed to mate for 4 h, and then separated again. Cohorts of 20 recently mated females were added to small container (10 × 10 × 20 cm) that was equipped with two Petri dishes (diameter, 5 cm) containing agar (1%), of which one was loaded with the odor in solvent, and the other with solvent only (or with another odor, if, e.g., when odors of infected vs. healthy flies were tested). Experiments were carried out in a climate chamber (25 °C, 70% humidity, 12 h light:12 h dark cycle). We added 50 µl of body wash extracts collected from either healthy (mock infection with LB media) or body washes from flies infected with *P. entomophila* for sequential time intervals. Flies were allowed to lay eggs for 3 days, after which eggs were counted to generate the oviposition indices (which were calculated as (*T* − *C*)/(*T* + *C*) where *T* is the number of eggs on the treatment plate and *C* is the number of eggs on the control plate).

### Courtship and mating experiments for single pairs

Adults were collected as newly emerged virgins, where males were kept in individually separated vials and females were reared in groups of 20–30 flies. Courtship was conducted with virgin flies that were 4–5 days old, and the behavioral experiments were conducted as described previously within the lid of an Eppendorf that was covered by a plastic slide^35^. Mating and courtship behaviors were recorded for 20 min and then analyzed. Copulation latency refers to the time delay until the successful physical coupling of the male and female, while copulation success refers to the percentage of total pairs that mated within the 60 min timespan. Copulation duration was the time that the male and female were conjoined during mating.

### Statistics and figure preparation

Statistical analyses were conducted using GraphPad InStat 3 (https://www.graphpad.com/scientific-software/instat/), while figures were organized and prepared using R Studio, Microsoft Excel, and Adobe Illustrator CS5. The Wilks–Shapiro test was used to determine normality of each data set. Normally distributed data were then analyzed using two-tailed, paired *t*-tests and one-way analyses of variance. Nonparametric distributed data were assessed using Kruskal–Wallis with Dunn’s post hoc test for multiple comparisons for selected pairs. An asterisk denotes statistical significance between two groups (*P ≤ 0.05, **P ≤ 0.01, ***P ≤ 0.001). Courtship data were analyzed and confirmed by an additional blind observer who was not aware of the treatments being viewed. Boxplots represent the median (bold black line), quartiles (boxes), as well as the confidence intervals (whiskers). Whiskers in barplots represent the standard error.

### Data availability

Additional supplementary information and extended data including methodology, courtship videos, and other raw data are available with the online version of the publication. All data supporting the findings of this study are available within the article and its Supplementary Information files.

## Electronic supplementary material

Supplementary Information
