# Supplementary material for: Pathogenic bacteria enhance dispersal through alteration of Drosophila social communication
Source: Nat Commun. 2017 Aug 16;8:265. doi: 10.1038/s41467-017-00334-9 (PMC5559524; doi:10.1038/s41467-017-00334-9)
Supplement: Supplementary file 1 — Supplementary Information [file 41467_2017_334_MOESM1_ESM.pdf]

File Name: Supplementary Information
Description: Supplementary Figures.

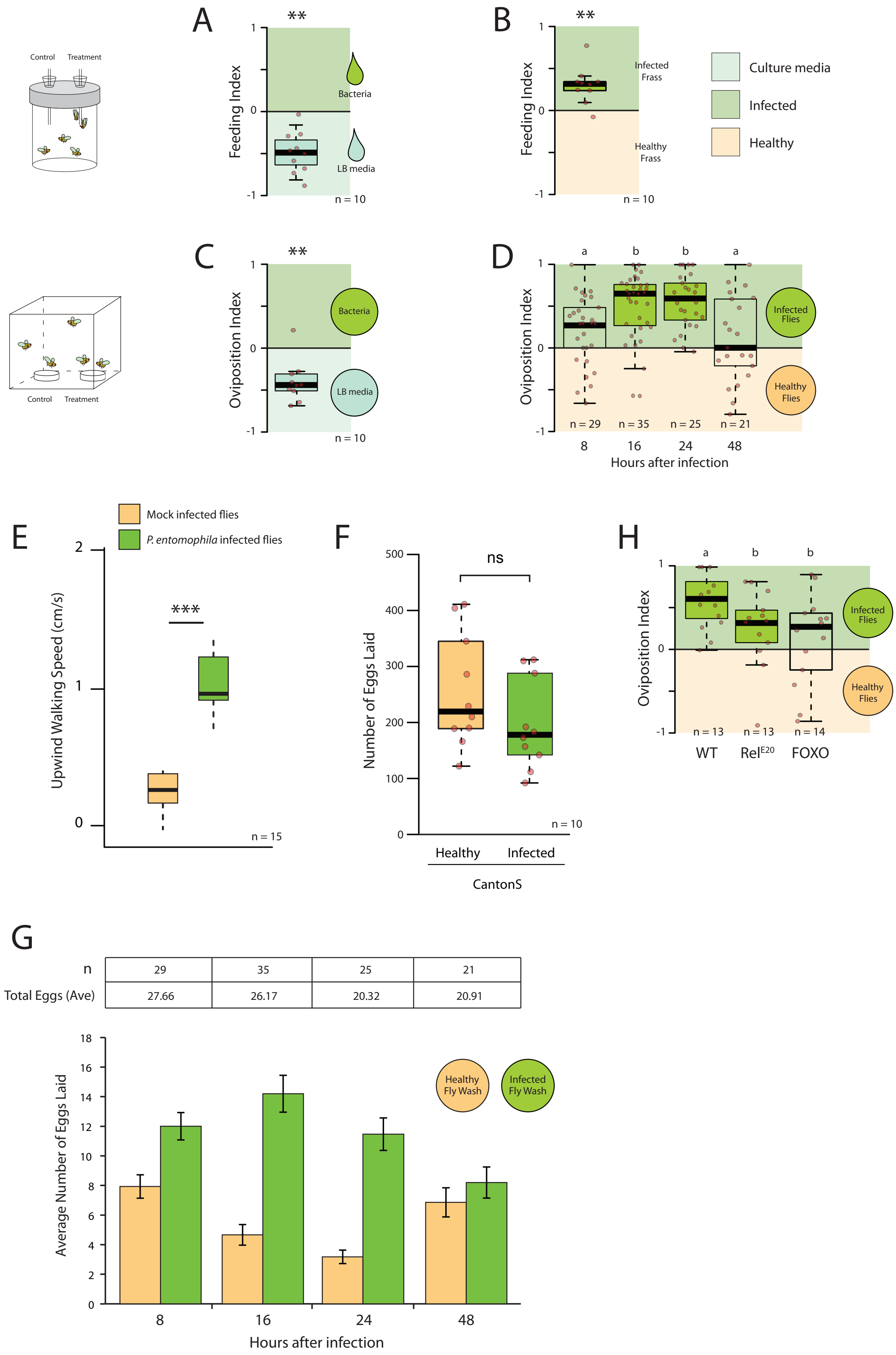
A
B
C
D
E
F
G
H
Control
Treatment
Feeding Index
Bacteria
LB media
n = 10
Infected Frass
Healthy Frass
Culture media
Infected
Healthy
Oviposition Index
Infected Flies
Healthy Flies
n = 29
n = 35
n = 25
n = 21
Hours after infection
Mock infected flies
P. entomophila infected flies
Upwind Walking Speed (cm/s)
n = 15
Number of Eggs Laid
ns
Healthy
Infected
CantonS
WT
RelE20
FOXO
n = 13
n = 14
n: 29, 35, 25, 21
Total Eggs (Ave): 27.66, 26.17, 20.32, 20.91
Average Number of Eggs Laid
Healthy Fly Wash
Infected Fly Wash

Supplementary Figure 1. Feeding and oviposition preferences towards infected flies and their frass. A, Feeding (**A**)and oviposition (**C**) preference of flies when given the choice between sugar solutions or agar plates containing growth media, or the media plus *Pseudomonas entomophila* bacteria (for definition of indeces see Fig. 1 of the manuscript). Feeding (**B**)and oviposition (**D**) preference of flies when given the choice between sugar solutions or agar plates containing frass of healthy or infected flies. Whenever time course is not shown, data were collected 24 hours after infection. **E**, Average upwind speed of flies after being exposed to a 1s-pulse of headspace of healthy or infected flies. For details of Flywalk assay see method part. **F**, Average number of eggs laid by each 20 healthy or infected female *Drosophila* during 24 hours. **G**, Average number of eggs laid by wildtype flies given a choice between the body washes of healthy and infected flies collected at different time points after infection (see S.Fig. 1D). Only 16 and 24 hour post-infection body washes generated a difference in egg-laying preference, but no difference in total number of eggs. **H**, Oviposition indices of naïve females towards body washes from wildtpe (WT), immune mutants (RelE20) or towards metabolic mutants (FOXO) after infection. Filled boxes are significantly different from zero, boxes with different letters differ significantly. In all box plots, filled boxes denote significance from zero.

A

Peak No.	Kovats Index	Compound Name	Female		Male	
			Healthy	Infected	Healthy	Infected
1	1320	Bromodecane (internal standard)	4.3	4.1	3.4	3.8
2	1485	Methyl laurate (ML)	0.3	24.9	0.3	3.5
3	1500	Lauric Acid	0.2	2.5	0	0.8
4	1710	Methyl Myristate (MM)	0.9	35.1	1	9.5
5	1725	Myrisitic acid	0	6.5	0	2.9
6	1900	Methyl palmitoleate	0.85	35.5	1.6	13.9
7	1912	Methyl palmitate (MP)	0.59	35.3	0.8	9.7
8	1920	Palmitoleic acid	0	3.4	0	2.3
9	1930	Palmitic acid	0	6.7	0	3.6
10	1969	Methyl linoleate	0.52	19.9	1.9	18.8
11	1980	Methyl oleate	0.67	30.2	1.4	16.6
12	1995	Methyl stearate	0	6.3	0	1.5
13	1998	Oleic acid	0	1.2	0	2.1
14	2210	Z-11-*cis*-Vaccenyl actetate (cVA)	0	0	14.2	9.9
15	2284	9-Z-Tricosene	0.68	1.4	6.3	6.1
16	2295	Heneicosane	0.84	2.6	5.2	5.5
			n = 3	n = 3	n = 3	n = 3

B

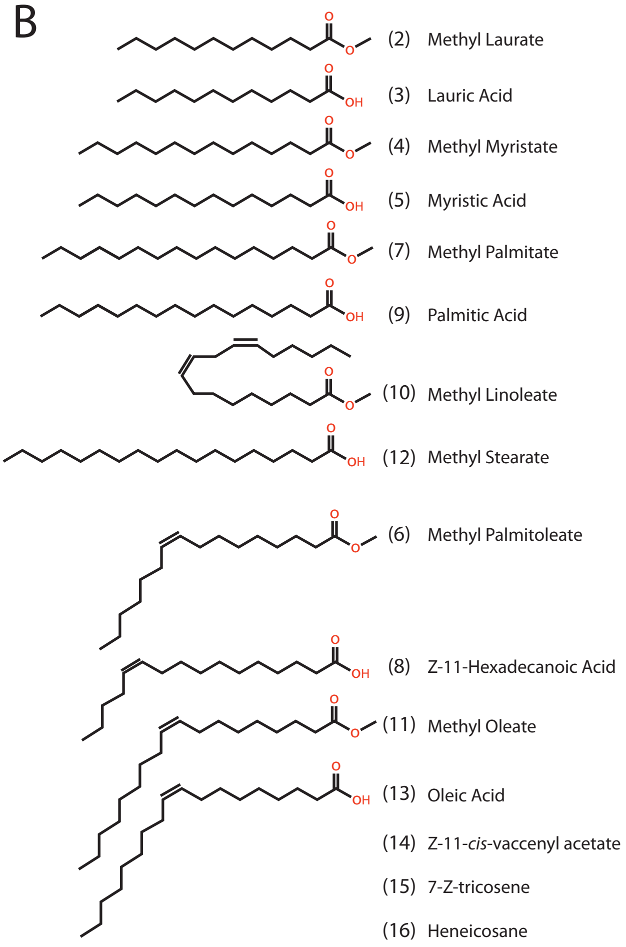

C

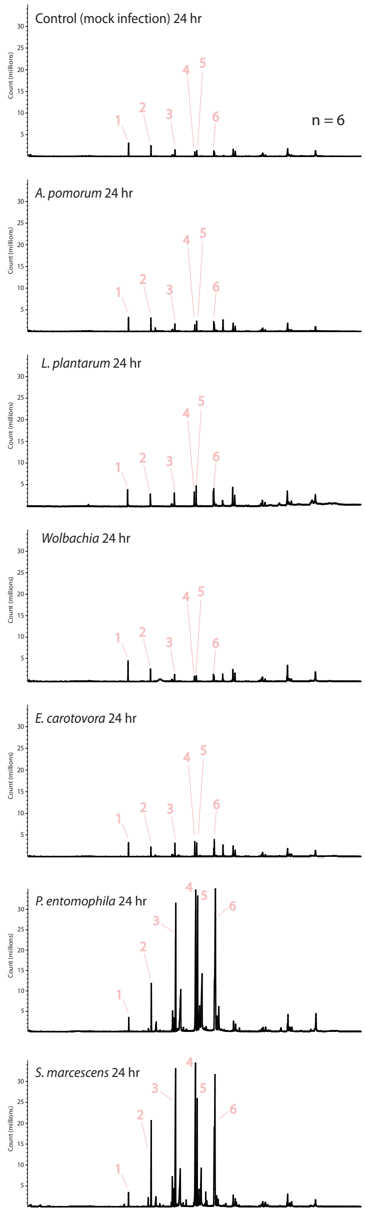

D

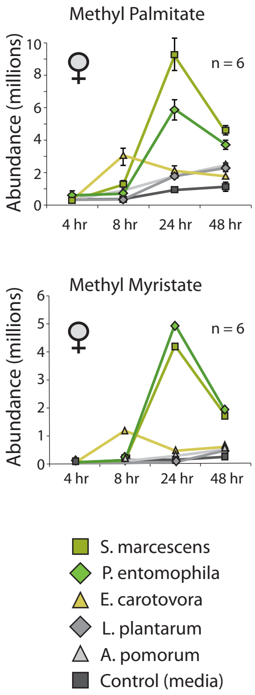

Supplementary Figure 2. GC-MS analyses of flies infected with pathogenic bacteria. **A**, Table corresponds to Fig. 1A, and shows the average amount of compounds identified from male and female flies after infection by *Pseudomonas entomophila* bacteria. The 12 colored compounds were those that were significantly different from healthy flies (Fig. 1I; ALL), while compounds in black were not different between healthy and infected flies. Red indicates the three methyl esters (3MEs), blue are the four fatty acids (4FAs) from Fig. 1I. **B**, Chemical structure of compounds identified from GC-MS data in Fig. 1A and B. **C**, Examples of raw total ion traces from adult female *Drosophila* after 24 hours of infection with several bacterial strains (Wildtype control (mock infected), *A. pomorum*, *L. plantarum*, *Wolbachia*, *E. Carotovora*, *P. entomophila*, *S. marcescens*). (1- bromodecane; 2- methyl laurate; 3- methyl myristate; 4- methyl palmitoleate; 5- methyl palmitate; 6- methyl oleate). **D**, Specific pheromone production over time for females infected with several strains of pathogenic and non-pathogenic bacteria.

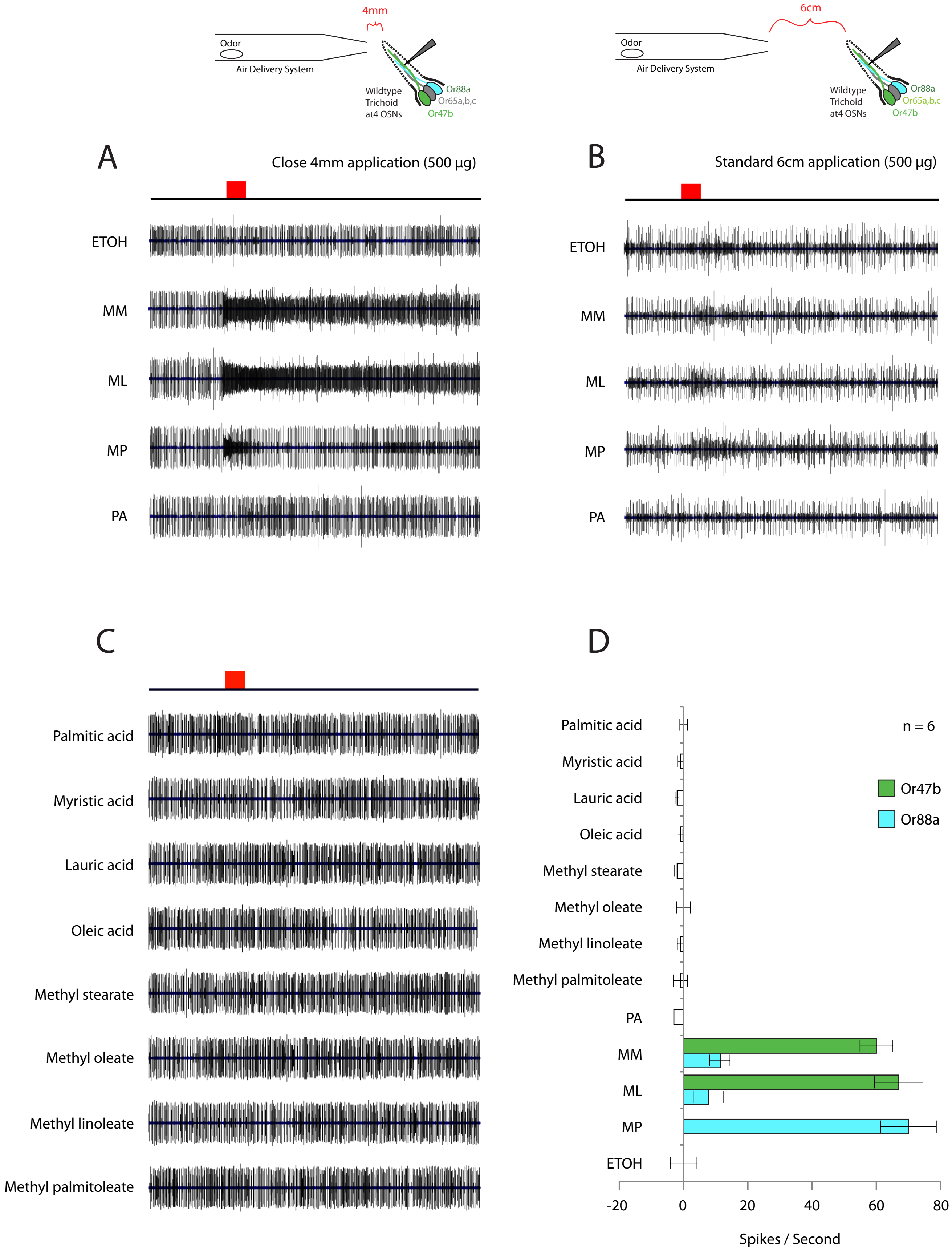

4mm
Odor
Air Delivery System
Wildtype
Trichoid
at4 OSNs
Or88a
Or65a,b,c
Or47b
6cm
Odor
Air Delivery System
Wildtype
Trichoid
at4 OSNs
Or88a
Or65a,b,c
Or47b
A
Close 4mm application (500 µg)
ETOH
MM
ML
MP
PA
B
Standard 6cm application (500 µg)
ETOH
MM
ML
MP
PA
C
Palmitic acid
Myristic acid
Lauric acid
Oleic acid
Methyl stearate
Methyl oleate
Methyl linoleate
Methyl palmitoleate
D
Palmitic acid
Myristic acid
Lauric acid
Oleic acid
Methyl stearate
Methyl oleate
Methyl linoleate
Methyl palmitoleate
PA
MM
ML
MP
ETOH
n = 6
Or47b
Or88a
-20
0
20
40
60
80
Spikes / Second

Supplementary Figure 3. Representative SSR traces for at4 responses to all odors that were increased after infection. A, *D. melanogaster* antennal trichoid 4 (at4) responses to the application of odors from 4mm cm distance (following the stimulus protocol of Lin et al. 2016), including ethanol solvent control (ETOH), and responses towards potential pheromone components that were upregulated after infection by *P. entomophila* (methyl myristate (MM), methyl laurate (ML), methyl palmitate (MP), and palmitoleic acid (PA). Large spike amplitudes correspond to the activation of the at4A neuron expressing Or47b, while smaller spikes correspond to the activation of the at4C neuron expressing Or88a. **B,** at4 responses to the application of odors from 6 cm distance (following the stimulus protocol of Dweck et al. 2015). **C**, SSR stimulation of at4 sensillum with other odors that increased after infection with *P. entomophila* bacteria. No responses were found for any of these odors. **D**, Quantified SSR responses towards 12 odors that were increased after infection and used in SSR testing of at4 OSNs.

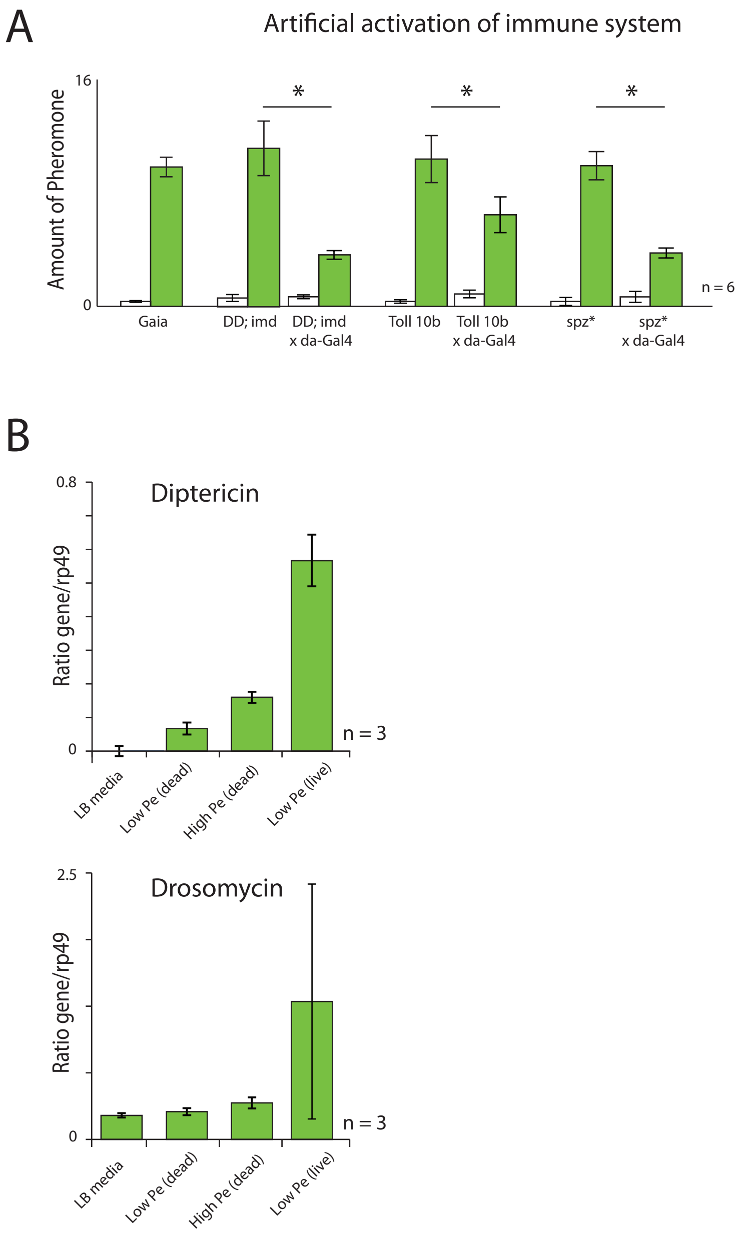
A
Artificial activation of immune system
Amount of Pheromone
16
0
*
*
*
n = 6
Gaia
DD; imd
DD; imd
x da-Gal4
Toll 10b
Toll 10b
x da-Gal4
spz*
spz*
x da-Gal4
B
0.8
Diptericin
Ratio gene/rp49
0
n = 3
LB media
Low Pe (dead)
High Pe (dead)
Low Pe (live)
2.5
Drosomycin
Ratio gene/rp49
0
n = 3
LB media
Low Pe (dead)
High Pe (dead)
Low Pe (live)

Supplementary Figure 4. Antimicrobial peptide (AMP) production after infection.
A, Artificial activation of Imd, Toll and Spatzle transgenic fly lines, showing that both immune response pathways are necessary (decreased after *P. entomophila* infection) but not sufficient (activation without infection) to observe maximum pheromone production following infection. **B**, Levels of two antimicrobial peptides in mock infected flies as well as in flies that were infected with different amount of living and heat-killed *P. entomophila*.

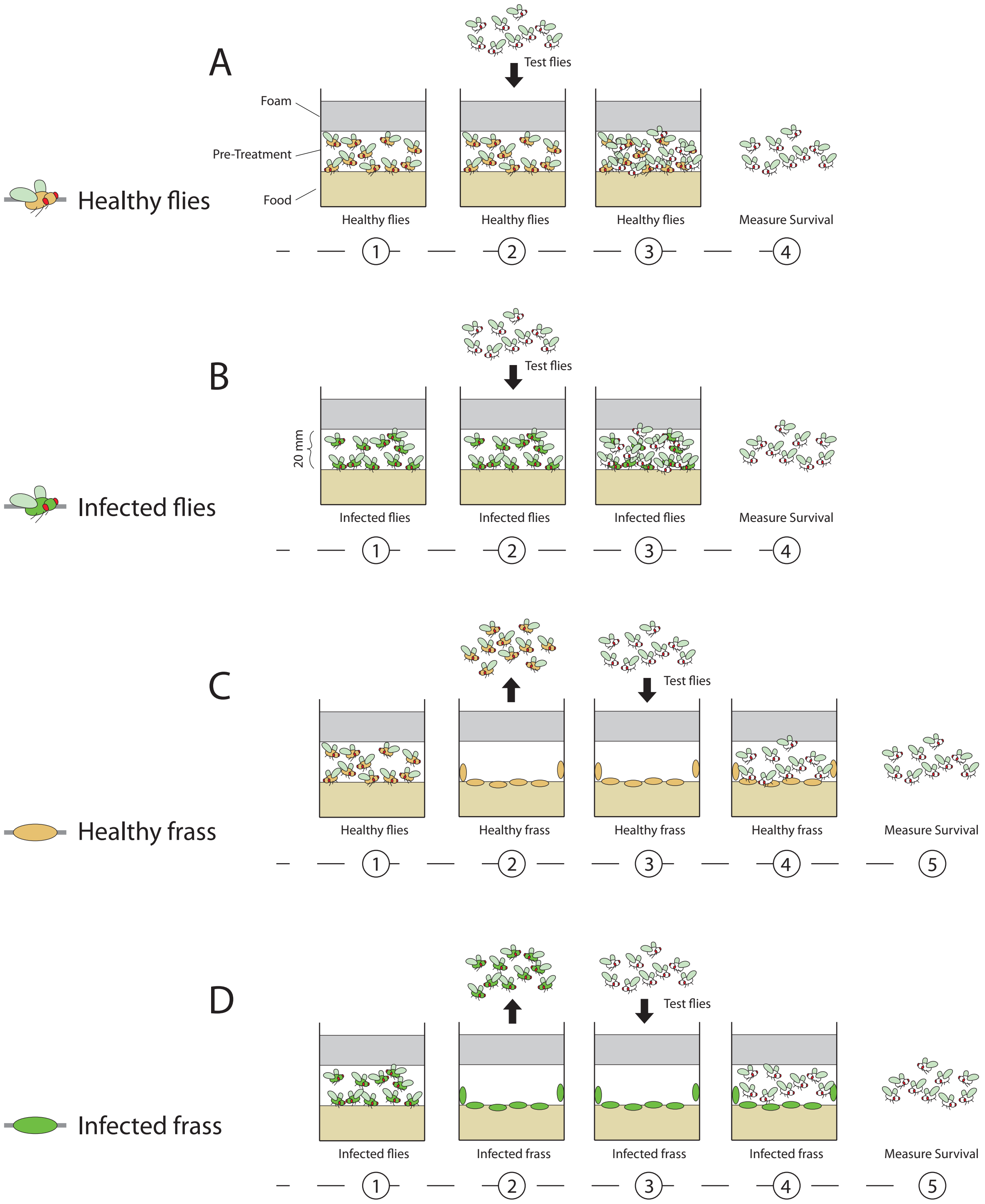

A
Test flies
Foam
Pre-Treatment
Food
Healthy flies
Healthy flies
Healthy flies
Healthy flies
Measure Survival
1
2
3
4
B
Test flies
20 mm
Infected flies
Infected flies
Infected flies
Infected flies
Measure Survival
1
2
3
4
C
Test flies
Healthy frass
Healthy flies
Healthy frass
Healthy frass
Healthy frass
Measure Survival
1
2
3
4
5
D
Test flies
Infected frass
Infected flies
Infected frass
Infected frass
Infected frass
Measure Survival
1
2
3
4
5

Supplementary Figure 5. Ecological consequences of attraction to sites of infection. Diagrams of the methodology for measuring the survival of cohorts of naïve flies that were placed in rearing vials that had previously been exposed to either healthy or infected adults and/or frass. **A**, healthy fly exposure to healthy flies. **B**, healthy fly exposure to infected flies. **C**, healthy fly exposure to healthy frass. **D**, healthy fly exposure to infected frass.
